# Melatonin confers fenugreek tolerance to salinity stress by stimulating the biosynthesis processes of enzymatic, non-enzymatic antioxidants, and diosgenin content

**DOI:** 10.3389/fpls.2022.890613

**Published:** 2022-08-08

**Authors:** Maryam Mohamadi Esboei, Amin Ebrahimi, Mohamad Reza Amerian, Hadi Alipour

**Affiliations:** ^1^Department of Agronomy and Plant Breeding, Faculty of Agriculture, Shahrood University of Technology, Semnan, Iran; ^2^Department of Plant Production and Genetics, Faculty of Agriculture, Urmia University, Urmia, Iran

**Keywords:** salinity stress, melatonin, physiological changes, fenugreek and diosgenin

## Abstract

Salinity-induced stress is widely considered a main plant-growth-limiting factor. The positive effects of melatonin in modulating abiotic stresses have led this hormone to be referred to as a growth regulator in plants. This study aims to show how melatonin protects fenugreek against the negative effects of salt stress. Different amounts of melatonin (30, 60, and 90 ppm), salinity stress (150 mM and 300 mM), and the use of both salinity and melatonin were used as treatments. The results showed that applying different melatonin levels to salinity-treated fenugreek plants effectively prevented the degradation of chlorophyll a, chlorophyll b, total chlorophyll, and carotenoid contents compared with salinity treatment without melatonin application. Besides, melatonin increases the biosynthesis of enzymatic and non-enzymatic antioxidants, thereby adjusting the content of reactive oxygen species, free radicals, electrolyte leakage, and malondialdehyde content. It was observed that applying melatonin increased the activity of potassium-carrying channels leading to the maintenance of ionic homeostasis and increased intracellular water content under salinity stress. The results revealed that melatonin activates the defense signaling pathways in fenugreek through the nitric oxide, auxin, and abscisic acid-dependent pathways. Melatonin, in a similar vein, increased the expression of genes involved in the biosynthesis pathway of diosgenin, a highly important steroidal sapogenin in medical and food industries, and hence the diosgenin content. When 150 mM salinity stress and 60 ppm melatonin were coupled, the diosgenin concentration rose by more than 5.5 times compared to the control condition. In conclusion, our findings demonstrate the potential of melatonin to enhance the plant tolerance to salinity stress by stimulating biochemical and physiological changes.

## Introduction

Plants normally face various biotic and abiotic stresses during their growth period, which may affect the quality and quantity of crops and prevent the plants from reaching their maximum genetic potential to achieve high yields (Parihar et al., 2015; Pandey et al., 2017). Salinity stress is one of the most major issues restricting plant production across the globe, and it has devastating environmental consequences (Kumar and Saddhe, 2018; Isayenkov and Maathuis, 2019). It affects plant growth, development, and productivity *via* ion toxicity, poor water absorption, and hormonal disorders (Ahanger et al., 2017; Kumar et al., 2020). Developing the plant species which are more tolerant to this environmental stress would be one of the most advantageous approaches to dealing with the unavoidable damages arisen out of salinity (Nxele et al., 2017; Kumar et al., 2020). Achieving such goal by applying methods based on classical plant breeding or biotechnology generally requires a deep understanding of plants’ physiological changes in response of plants to salinity stress (Parihar et al., 2015; Hanin et al., 2016).

**Figure 1:**
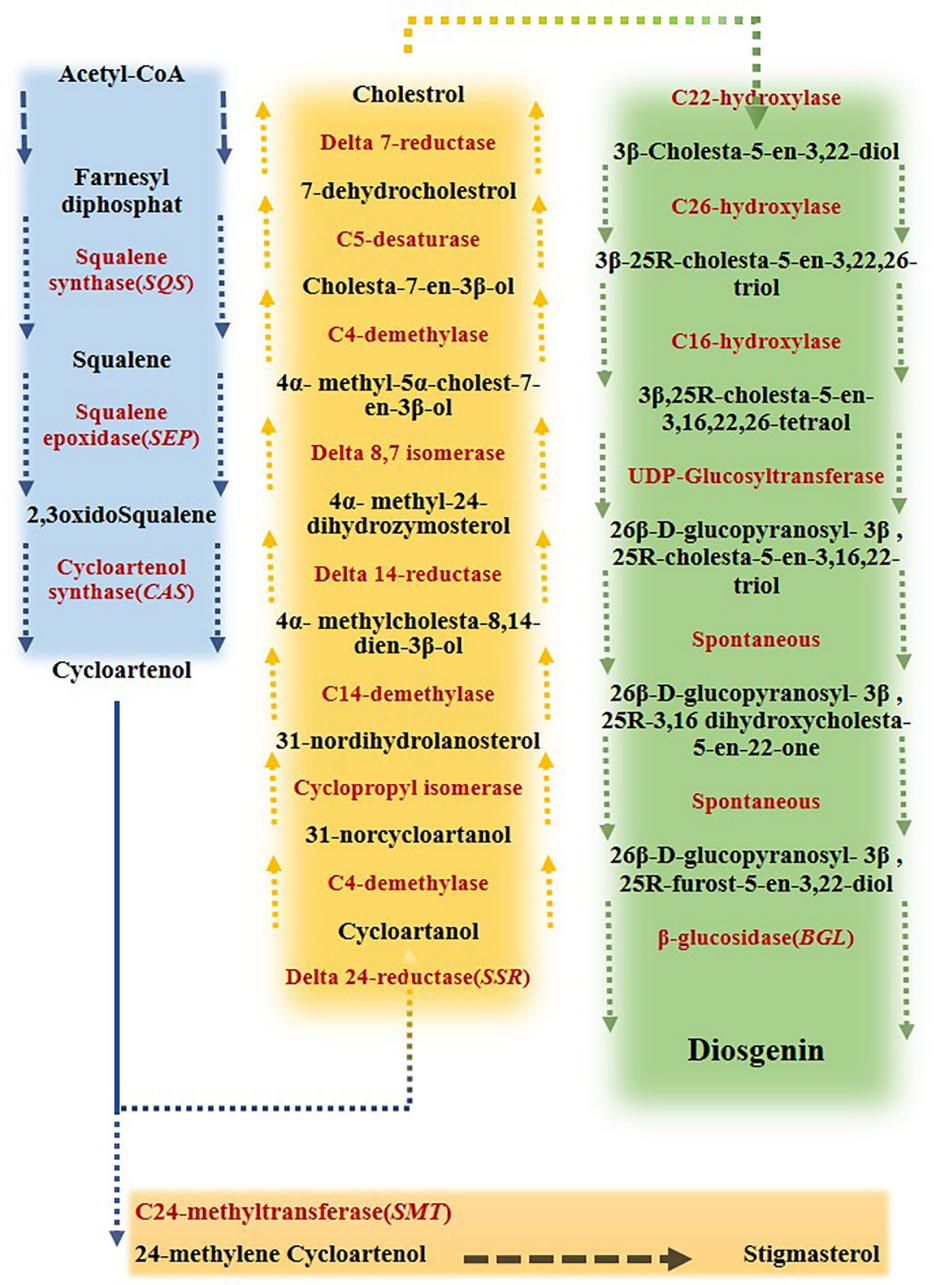
Biosynthesis pathway of diosgenin from farnesyl diphosphate in fenugreek. Acetyl-coenzyme A (acetyl-CoA) converts to cycloartenol via a series of multiple reactions. Cycloartenol converts to either cycloartenol, leading to cholesterol formation, or 24 methylene cycoartanol, leading to stigmasterol. This branch-point has a great influence on disogenin biosynthesis. Cholesterol then converts to 3ß -cholesta- 5-en- 3, 22-diol, which ultimately results in diosgenin production (Mohammadi et al., 2020).

Fenugreek (*Trigonella foenum-graecum*) is a dicotyledonous annual plant belonging to the Fabaceae family, the subfamily of Papilionaceae, is commonly used not only as an edible vegetable but also as a raw material for some kinds of medicine. In terms of medicinal use, fenugreek is of great importance as it contains diosgenin (McAnuff et al., 2005; Wani and Kumar, 2018). This valuable compound, with a molecular formula of C_27_H_42_O_3_, generally forms an amorphous powder and benefits from a unique chemical and thermal stability under a wide range of conditions (Blunden and Rhodes, 1968). Diosgenin, as a valuable component of steroid drugs, such as testosterone, progesterone and glucocorticoids, could be quite helpful in treating cholesterol, hyperlipidemia, diabetes, metabolic diseases, cancer and aging (Upadhyay et al., 2014; Wani and Kumar, 2018). Two different pathways were suggested by the literature review for diosgenin production, including lanosterol (LAN) to cholesterol and cycloartenol to cholesterol pathways. It has been known that some key genes direct phytosterols biosynthesis pathways (He et al., 2008; Vaidya et al., 2013; Gas-Pascual et al., 2014; Cheng et al., 2021). The squalene, produced *via* converting two molecules of farnesyl pyrophosphate by Squalene synthase (*SQS*), is oxidized to oxidosqualene by Squalene epoxidase (*SEP*). Next, cycloartenol is generated *via* oxidization of oxidosqualene by cycloartenol synthase (*CAS*). Then, cholesterol and phytosterols such as sitosterol are formed by the activities of Δ24-reductase sterol- methyltransferase (*SMT1*), respectively. Finally, furostanol 26-O-beta-glucoside is deglycosidased by furostanol glycoside 26-O-beta-glucosidase (*BGL*) and transformed to diosgenin (He et al., 2008; Vaidya et al., 2013; Gas-Pascual et al., 2014). Eventually, after a few steps, furostanol glycoside 26-O-beta-glucosidase catalyzes the final step of diosgenin formation, which deglycosidases the furostanol 26-O-beta-glucoside to diosgenin (Figure 1; He et al., 2008; Vaidya et al., 2013; Gas-Pascual et al., 2014; Mohammadi et al., 2020).

Recent studies implied that the adverse impacts of abiotic stresses on plants might be largely modified through the exogenous application of various elicitors, like osmoprotectants (Wang et al., 2017; Estaji et al., 2019), phytohormones (Sytar et al., 2019; Rhaman et al., 2020), polyamines (Liu et al., 2016; El Amrani et al., 2019), and cold plasma (Ling et al., 2015; Ebrahimibasabi et al., 2020). Melatonin (N-acetyl-5-methoxytryptamine) is a natural antioxidant generated by all living species, including microbes, plants, and animals (Dubbels et al., 1995; Zhao et al., 2019). This chemical regulates flowering and seed germination, plant growth and development, photosynthesis, and enhancing stress tolerance in plants (Nawaz et al., 2016; Bose and Howlader, 2020).

Melatonin has been dubbed a “new growth regulator” in plants due to its favorable effects in moderating biotic and abiotic stresses (Arnao and Hernández-Ruiz, 2015; Al-Huqail et al., 2020; Bose and Howlader, 2020; Siddiqui et al., 2020a). Melatonin has a much higher antioxidant capacity than other antioxidants, which is probably related to its efficient transport *via* cell pores (Tan et al., 2002; Reiter et al., 2013). In addition to its antioxidant activity, the melatonin regulates the expression of many genes involved in physiological and biochemical processes (Siddiqui et al., 2019, 2020b; Bose and Howlader, 2020). Melatonin has been repeatedly mentioned as an important factor in improving plant response to drought (Ye et al., 2016; Li et al., 2018), salinity (Li et al., 2017a; Ren et al., 2020), cold stress (Yang et al., 2018b), herbicide protection (Park et al., 2012), ion toxicity (Yadu et al., 2018; Al-Huqail et al., 2020), and oxidative stress (Park et al., 2012).

In previous studies, the adverse effects of salinity stress on the quality and quantity of fenugreek were well established, although salinity has effectively increased the content of some important secondary metabolites, such as diosgenin and trigonelline in fenugreek (Sahari et al., 2016; Noohpisheh et al., 2021; Banakar et al., 2022). Environmental stresses on medicinal plants might vary from those on other plants, particularly agricultural crops. Although abiotic stress causes a decline in production in medicinal plants, the concentration of secondary metabolites, which are the most useful medicinal chemicals, eventually rises (Yang et al., 2018a; Li et al., 2020; Ogbe et al., 2020). In general, there are few reports on the melatonin effects on enhancing fenugreek tolerance under abiotic stresses and changing the secondary metabolites content, as well as the interaction effects of abiotic stresses and melatonin as two important elicitors on the secondary metabolite content in fenugreek (Xalxo and Keshavkant, 2019; Zamani et al., 2020). Current study aimed to assess the efficiency of melatonin in improving tolerance to salinity stress and its effect on the diosgenin content in fenugreek. Furthermore, identifying the optimal concentration of salinity and melatonin stress to increase the diosgenin content in fenugreek was another goal of this study.

## Materials and methods

### Plant materials and treatments

First, the seeds of fenugreek (Boshruyeh genotype) surface were sterilized in a 1% (v/v) sodium hypochlorite solution for 10 min, then the seeds were rinsed several times with sterile water and sown in the 25^*^35 cm pots containing a combination of peat moss, perlite, and sand in equal proportions in a growth chamber with a photoperiod of 16–8 h at a (day: night) temperature of 25–22°C with 60–70% humidity. In order to provide the plants with light, fluorescent lamps with a light intensity of the 400 μmol m^−2^ s^−1^ were used. The experimental treatments included control (the plants without melatonin and salinity) and the plants treated with different levels of melatonin (0, 30, 60, and 90 ppm), salinity (0, 150, and 300 mM), and the simultaneous use of salinity and melatonin. These concentrations were selected based on preliminary experiments. To prepare the melatonin solutions, the solute was dissolved in ethanol and then, distilled water was used to dilute the compound [ethanol/water (v/v) = 1/10000]. After establishing the establishment of seedlings at the three-leaf stage, various doses of melatonin (30, 60, and 90 ppm) were put to the irrigation water and added to the pots for 7 days. Then, a combination of ethanol and distilled water in a ratio of 1 in 10,000 was used to irrigate the control group (no melatonin). Next, 15 days of salinity treatments were administered. The plants were randomly divided into 12 groups: control, control plus 30, 60, and 90 ppm melatonin, salinity stress (150 mM), salinity stress (300 mM), salinity stress (150 mM) plus melatonin (30,60, and 90 ppm), and salinity stress (300 mM) plus melatonin (30,60, and 90 ppm). Salt and melatonin solution were added to each pot exactly equal. Finally, leaf samples (5 week old plants) were collected and stored in a freezer at −80°C. Melatonin and NaCl used in this study were purchased from Sigma-Merck Company (EC number: 200–797-7, CAS number: 73–31-4; EC number: 231–598-3, CAS number: 7647-14-5, respectively). According to the treatments applied, this study was conducted as a factorial in a completely randomized design with three replications.

### Pigments measurement

First, 500 mg of leaf tissue was thoroughly ground with liquid nitrogen and mixed with 10 ml of dimethyl sulfoxide (Arnon, 1967). After heating the solutions in a water bath at 75°C for 4 h, the samples were centrifuged at 3000 rpm for 30 min at 4°C. Lastly, by using a spectrophotometer (UV-1800; Shimadzu Corporation, Kyoto, Japan), the amount of light absorption of supernatant was identified at wavelengths of 480, 649 and 665 nm and the traits were calculated based on the following formulas:


Chlorophylla=12.25A665–2.79A649

Chlorophyllb=21.5A649–5.1A665

$$\mathrm{Total carotenoids}=\left(1000A480–1.82\mathrm{Cha}-85.02\mathrm{Chb}\right)/198$$

### Relative water content

According to approach developed by Kirnak et al. (2001), with the aim of measuring relative water content, the fresh weight of five selected leaves from each plant were measured. After keeping the leaves immersed in distilled water for a whole night at 4°C with low light, the plant samples were transferred and kept in an oven for 24 h. By determining the weight of the entire dried plant leaves, their relative water content was calculated based on the following formula:


$$\%\mathrm{Water content}=\left[\left(\mathrm{Wf}-\mathrm{Wd}\right)/\left(\mathrm{Wt}-\mathrm{Wd}\right)\right]\;\;\times \;\;100$$

In this formula, Wf is fresh leaf weight, Wt is swollen leaf weight and Wd is leaf dry weight.

### Soluble protein content and antioxidant activities

To extract the enzymatic solutions, 250 mg of finely crushed plant material (in a mortar with liquid nitrogen) was added to falcon tubes containing 2.5 ml of phosphate buffer (pH = 7.0, 50 mM) each. After vortexing the samples, they were centrifuged at 1500 rpm for 15 min at 4°C. The supernatant was transferred to separate tubes and was used to measure the activity of catalase (CAT), ascorbate peroxidase (APX), guaiacol peroxidase (GPX), superoxide dismutase (SOD), polyphenol oxidase (POD) and soluble protein. Total protein was measured by the Bradford (1976) method using a spectrophotometer (UV-1800; Shimadzu Corporation, Kyoto, Japan). Catalase activity was measured according to Scebba et al. (1998), which was based on the breakdown of hydrogen peroxide by the catalase enzyme. The activity of superoxide dismutase was measured spectrophotometrically (UV-1800; Shimadzu Corporation, Kyoto, Japan) and based on its inhibitory ability to photochemically reduce the nitrobluetetrazolium (NBT) at a wavelength of 560 nm (Kono, 1978). The activity of guaiacol peroxidase was measured at 470 nm (Drotar et al., 1985). The activity of ascorbate peroxidase and polyphenol oxidase was measured according to Ranieri et al. (2000), and Gauillard et al. (1993), respectively. Due to the reaction between ascorbate peroxidase and ascorbic acid and hydrogen peroxide, dehydro ascorbate is produced, which is read at a wavelength of 290 nm. The mixing compound contains 950 μl of 0.1 ml EDA, 1600 μl of 50 ml phosphate buffer (pH = 7), 400 μl of ascorbic acid 0.5 mM, 10 μl of hydrogen peroxide (30%) and 50 μl of antioxidant enzyme extract.

### Electrolyte leakage

The unimpaired leaves were identified and separated from all treatments, and using the punching machines, the squared leaves (1*1 cm) were achieved and were subjected to 10 ml of distilled water in the falcon tubes. Then, the mixtures were well shaken for 30 min and by applying an EC meter (Weilheim, Germany) at a temperature fixed at 25°C, EC values (Ec1) were recorded. The falcon tubes and their contents were then kept in a water bath with a temperature of 90°C for 60 min prior to measuring their electrical conductivity (Ec2; Hepburn et al., 1986). Finally, using the mentioned method, the damage index was calculated based on the following formula:


$$\%\mathrm{EL}=\left(\mathrm{Ec}1/\mathrm{Ec2}\right)\;\;\times \;100$$

### Measurement of lipid peroxidation based on MDA

To analyze lipid peroxidation, 250 mg of ground leaf tissue (in a mortar with liquid nitrogen) was accurately weighted and then subjected to 5 ml of 50 mM potassium phosphate buffer (pH = 7.2). After vortexing, the solution was centrifuged at 3000 rpm for 15 min. Next, the supernatant and 0.5% thiobarbituric solution comprising 20% trichloroacetic acid (2 ml) was blended. The falcon tubes were exposed to a water bath at a temperature of 90°C for 30 min, immediately followed by an ice bath for the same time. The absorbance at 532 and 600 nm was measured using a spectrophotometer (UV-1800; Shimadzu Corporation, Kyoto, Japan; Stewart and Bewley, 1980) after 15 min of centrifugation at 3000 rpm. Using the following equation, the quantity of malondialdehyde was calculated:


$$\mathrm{MDA}=\left[\left(532\mathrm{nm}–600\mathrm{nm}\right)/\left(\mathrm{QD}\;\;\times \;\;\mathrm{QF}\right)\right]\;\;\times \;\;\mathrm{DF}$$

In this formula, QD = Cuvette diameter (1 cm), QF = Extinction coefficient (155 mmol/cm) and DF = Dilution factor (20).

### Total phenol and flavonoid content

By subjecting the plant samples to a temperature of 40°C prior to pulverization, they were thoroughly dried. After that, the samples were subjected to10 ml of 80% methanol per gram, and the achieved solution was shaken for a whole night. Then, the solutions were passed *via* a filter paper and the content of phenol and total flavonoid in the final extract was determined (McDonald et al., 2001). A mixture of 0.5 ml of folin–ciocalteu reagent (10%) and 4 ml of Na_2_CO_3_ solution (1 M) was blended with the 0.5 ml of plant extract, kept at room temperature for 15 min, and was measured using a spectrophotometer (UV-1800; Shimadzu Corporation, Kyoto, Japan) at 765 nm. Three replications were applied for all samples and standard curve of gallic acid (Merck, CAS Number: 149–91-7, EC Number: 205–749-9) at concentrations of 0, 20, 40, 60, 80, 100, 120 and 140 mg/l was used to determine the content of total phenol. Based on the aluminum chloride colorimetric approach, the total flavonoid content was measured. For this purpose, 400 μl of 2% aluminum chloride in liquid form was thoroughly mixed with 100 μl of sample solution. After introducing 1,200 μl of potassium acetate solution to the mixture and keeping it at 37°C for 40 min, the absorbance of the samples was recorded at 415 nm (Quettier-Deleu et al., 2000). The samples were analyzed for their flavonoid content by plotting the standard curve of rutin (Merck, CAS Number: 250249–75-3, EC Number: 205–814-1) at concentrations of 0, 20, 40, 60, 80, 100, 120, and 140 mg/l.

### Total sugar content

The powdered leaf tissue was treated with 1.5 ml of 80% ethanol, vortexed for 10 min, and centrifuged at 3000 rpm for 15 min, and the supernatants were kept at 50°C to dry the existing alcohol. Prior to the second centrifugation, the dried samples were subjected to 10 ml of distilled water, 0.5 ml of normal barium hydroxide, and 0.5 ml of 5% zinc sulfate. 2 ml of the resultant supernatant was harvested and treated by 1 ml of 5% phenol and 5 ml of 98% sulfuric acid (V/V). After incubating the solutions at room temperature for 45 min, a spectrophotometer (UV-1800; Shimadzu Corporation, Kyoto, Japan) was applied to record the absorbance at 485 nm (Schlegel, 1956).

### Nitric oxide content

The instructions suggested by Zhou et al. (2005) were followed in determining nitric oxide content. Moreover, 0.5 g of finely ground leaf tissue was subjected to 3 ml of 50 mM cold acetic acid containing 4% zinc acetate (pH = 4). After performing the centrifugation at 3000 rpm for 15 min, the supernatant was harvested and washed with 1 ml of extraction buffer and underwent another round of centrifugation under the mentioned conditions. After thoroughly combining the supernatant with 0.1 g of activated charcoal, it was filtered. In the last stage, 1 ml of grease reagent was added to 1 ml of the solution, followed by 30 min of incubation at room temperature. At 540 nm, the adsorption rate of the solution was measured. NO content was determined with the NaNO_2_ standard curve (Sigma, USA, CAS Number: 7632-00-0, EC Number: 231–555-9).

### H_2_O_2_ content

First, 5 ml of 0.1% trichloroacetic acid (TCA) was well mixed with 0.5 g of the leaves, which were powdered in liquid nitrogen. The obtained mixture was subjected to centrifugation at 3500 rpm for 15 min. Then, the mixture was treated with 750 μl of supernatant, 750 μl of 10 mM potassium phosphate buffer (pH = 7.2), and 1.5 ml of potassium iodide (1 M), and the adsorption rate was recorded using a spectrophotometer (UV-1800; Shimadzu Corporation, Kyoto, Japan) at 390 nm. Finally, H_2_O_2_ content was plotted and measured using standard (Sigma, USA, CAS Number: 7722-84-1) curves (Loreto and Velikova, 2001).

### Endogenous melatonin content

To measure melatonin, 2 g of powdered leaves were treated with 10 ml of methanol and then ultrasonicated at 25°C for 35 min. After performing centrifugation at 4000 rpm at 4°C for 15 min, the supernatant was harvested and dried using liquid nitrogen. Then, the obtained powder was subjected to 2 ml of 0.5% methanol, and then passed *via* an 18C solid phase extraction column which was previously activated with methanol and water. After that, the resulting solution was passed *via* a 0.2 μm filter (Z290823, Sigma-Aldrich, Taufkirchen, Germany) and underwent analysis using an HPLC column (Agilent Technologies Inc., USA, 1200 series, C18, 4.6 μm, 250 mm length, 5 mm diameter) at a wavelength of 280 nm to determine the melatonin content. The HPLC system consisted of a reversed-phase Nucleosil-100 C18 column with the integrated precolumn guard, an isocratic HPLC pump. Consideration was given to the mobile phase, which consisted of acetonitrile, Na_2_HPO_4_/H_3_PO_4_ buffer (15:85, pH = 4.5; Pothinuch and Tongchitpakdee, 2011), and a 1 ml/min flow rate. The volume injection and retention time were 20 μl and approximately 10 min, respectively. To determine the sample’s melatonin concentration, the sample peak area was compared to the melatonin standard curve (Sigma, USA, CAS Number: 73–31-4, EC Number: 200–797-7). The samples were dissolved in methanol and then placed in ultrasonic to prepare 50, 100, 150, 300, 500, 750, and 1,000 ppm of melatonin. The correlation coefficient (*R*^2^) was calculated 0.985 for the data sets.

### Auxin content

For this purpose, 1.5 g of powdered leaves were thoroughly homogenized in 20 ml of an extract consisting of water and methanol in equal proportion. After 15 min of centrifugation at 3000 rpm, the solution was put in a freeze dryer to be chilled and evaporated, and 1 ml of 80% methanol was added. Prior to HPLC analysis (Agilent Technologies Inc., USA, 1200 series), the reconstituted eluate was filtered through a 0.45 m Whatman glass microfiber filter to remove impurities. The HPLC column was heated to 30°C with a 0.45% formic acid: acetonitrile gradient [0–5 min, 95:5% (v/v); 5–6 min, 95:5% to 0:100% (v/v); 6–16 min, 0:100% (v/v); Cao et al., 2006]. Eventually, the auxin concentration in leaf tissue was determined at the flow rate of 0.8 ml/min using an HPLC column (C18, 4.6 μm, 250 mm length, 5 mm diameter). The volume injection and retention were 20 μl and approximately 9 min, respectively. Peak spiking, retention time, and spectral properties were applied to identify the peak position. Furthermore, a linear regression equation of standard (Sigma, USA, CAS Number: 87–51-4, EC Number: 201–748-2) calibration curves was applied to determine the auxin concentration. The correlation coefficient (R^2^) was calculated 0.99 for the six different concentrations that were evaluated. The target component was quantified by the peak areas at the maximum wavelength of 260 nm.

### Content of abscisic acid

First, 2.0 g of fresh leaves were well ground and subjected to 1 ml of a solution composed of methanol, ethyl acetate, and acetic acid at the rate of 1:50:49. Then, 20 mg of butylated hydroxytoluene (BTH) was introduced to this solution as antioxidant (Hubick and Reid, 1980). The solution was then passed *via* a 0.45 μm Whatman glass microfiber filter and by adding distilled water, the final volume was raised to 100 ml. Under vacuum and at a temperature of 35°C, a rotary apparatus was applied with the aim of evaporating the solvent (Hubick and Reid, 1980). Then, the samples were exposed to 10 ml of 50 mM hydrogen phosphate (pH = 7) followed by filtration. After adjusting the pH to 4.2 (Dobrev and Kaminek, 2002). 10 ml of ethyl acetate was added to the solution. The solution was then treated with 0.1 g of sodium sulfate to dehydrate the abscisic acid-containing ethyl acetate solution. After adding 5 ml of methyl chloride to the dried extract in order to evaporate it, the solutions were incubated at room temperature for 24 h. Finally, the extract was combined with 300 l of a 1% acetic acid-containing methanol solution and filtered. A HPLC column (Agilent Technologies Inc., USA, 1200 series, C18, 4.2 μm, 250 mm length, and 5 mm diameter) was applied with an interval of 4 to 5 min based on standard abscisic acid to determine the concentration of this important compound. In chromatographic separation stage, two mobile phases, including a medium comprised of water/acetonitrile/formic (A) acid in the volume ratio of 94.9:5:0.1, respectively, and another medium comprised of the same materials in the volume ratio of 10:89.9:0.1 (B), respectively, were applied. The elution program maintained 100% A for 5 min, followed by two consecutive linear gradients from 0 to 6% B in 10 min, and from 6 to 100% B in 5 min, and finally 100% B maintained for another 5 min (Dobrev and Kaminek, 2002). A flow rate of 0.8 ml/min was applied at a wavelength of 254 nm. The volume injection and retention were 20 μl and approximately 11 min, respectively. Finally, quantification was carried out given the specific area of the peak in relation to the retention time of the standard abscisic acid sample. ABA standard was obtained from Sigma, USA (CAS Number: 21293–29-8, EC Number: 244–319-5). The correlation coefficient (R^2^) was calculated 0.98 for the six different concentrations that were evaluated. The target component was quantified by the peak areas at the maximum wavelength of 260 nm.

### Cl^−^ content

A total of 6 ml of a mixture of HNO_3_: HCl (5:1 V/V) was added to 0.2 g of the finely ground plant samples and well homogenized. It aimed at evaporating the free acid in the solution, the digested materials were kept at 95°C for 5 h in a water bath. Using ion-free distilled water, the solutions were passed through a filter paper to a volume of 50 ml (Sagner et al., 1998). Finally, utilizing an atomic absorption spectrometer (105,924 GBC sensAA), Cl^−^ concentration was determined.

### Potassium and sodium content

The plant leaves were dried at 70°C for 48 h, and 0.1 g of the powdered leaves was accurately weighted. Then, 10 ml of glycolic acetic acid (0.1 N) was added to the plant materials, and the falcons were left at ambient temperature for 24 h. Prior to filtering the samples, they were kept at 70°C for 2 hours. Using a flame photometer (Shannon, Co. Clare, Ireland, PFP7 incl), the filtered extract was used to determine the concentration of the specified components in the investigated plant (Isaac and Kerber, 1971).

### RNA extraction and cDNA synthesis

The RNA extraction process was carried out by following the manufacturer’s instructions accurately and utilizing an RNeasy plant mini kit (Qiagen). To remove DNA contamination, one microgram of RNA extract was treated with DNase I (Thermo Fisher Company). Furthermore, the RNA was assessed qualitatively and quantitatively applying 1% agarose gel and Nanodrop. Besides, 20 microliters of cDNA were synthesized using iScript cDNA synthesis kit (Bio-Rad, Hercules, CA, USA) according to the manufacturer’s instructions. The primers (Supplementary Table S1) were designed applying the Primer 3 Plus online program, and their accuracy was checked using Oligo Analyzer v.3.1.^1^

### Real-time PCR reaction

For real-time PCR reaction, SYBR^®^Green PCR Master Mix 2X (Ampliqon) was applied. The reactions in question were conducted in a volume of 10 μl with three biological replications. Besides, a negative control (containing the all reaction components except for cDNA) was applied during the whole experiment to ensure that the samples were not contaminated. The mentioned reaction was conducted by applying 35 cycles of 95°C for 20 S, and 62°C for 40 S preceded by 95°C for 15 min.

### Determining the diosgenin content

First, 1 g of powdered plant leaves was dissolved in 20 ml of 96% ethanol, and the mixture was thoroughly homogenized. Next, the solutions were ultrasonicated for 30 min and then, treated by 20 ml of sulfuric acid (2 N), and kept at 95°C for 2 h. Separating the solution ingredients by pure n-hexane was carried out three times. It aimed at neutralizing the acidic state, the solution was well washed with sodium hydroxide (1 M). The resulting solution was then washed with distilled water twice. Afterward, the n-hexane solution containing diosgenin was dried by subjecting it to a vacuum generated by a rotating equipment, and then dehydrated with 0.2 g of anhydrous sodium sulfate. After dissolving the obtained extract in acetonitrile, it was passed through a 0.22 μm Whatman glass microfiber filter (Zhou et al., 2010). The experimental conditions were isocratic binary system of acetonitrile: water (90:10). The process of determining the diosgenin content was carried out by utilizing a HPLC column (Agilent Technologies Inc., USA, 1200 series, C18, 4.2 μm, 250 mm length, 5 mm diameter) at the flow rate of 0.8 ml/min and the wavelength of 210 nm. Different concentrations of diosgenin (Sigma-Aldrich-Germany; CAS Number: 512049) including, 100, 150, 300, 500 and 1,000 ppm were produced by dissolving in acetonitrile prior to placing the samples in ultrasonic for 30 min. The volume injection and retention were 30 μl and approximately 23 min, respectively. Finally, the correlation coefficient (R^2^) was calculated 0.99 for the data sets.

### Data analysis

Regarding the specific nature of the experimental data, the present study was carried out based on a factorial experiment in a completely randomized design. Using SPSS version 26 (IBM SPSS, Armonk, NY, USA),^2^ Duncan’s approach was used to do an analysis of variance with a comparison of means. At the 1% probability level, both the simple and combined effects of salinity and melatonin on all physiological and biochemical parameters were significant. Supplementary Table S2 displays the findings of a variance analysis of physiological and biochemical characteristics. Furthermore, melting and amplification curve analysis were accurately carried out to confirm the accuracy of the data before analyzing the expression data of the genes. To analyze the data obtained from real-time PCR, the relative expression of each gene was measured according to the relative standard curve method based on the formula 2^−ΔΔCt^ (Livak and Schmittgen, 2001).

## Results

### The effects of melatonin on pigment and relative water contents under salt stress

The results showed that the contents of chlorophyll, carotenoids, and relative water significantly decreased (*p* < 0.01) under different salinity levels compared to the control. This reduction in chlorophyll a, chlorophyll b, total chlorophyll, carotenoids, and relative water contents at 300 mM salinity were 60, 56, 59, 55, and 53% respectively, compared to the control. Applying different levels of melatonin (especially, 30 and 60 ppm) in the absence of salinity stress led to a significant rise in these traits contents compared to the control (Table 1).

**Table 1:** The mean comparison effects of melatonin concentrations and different levels of NaCl on pigments and relative water content.

Treatment		Chlorophyll a (mg g^−1^ FW)	Chlorophyll b (mg g^−1^ FW)	Total chlorophyll (mg g^−1^ FW)	Carotenoids (mg g^−1^ FW)	Relative water content (%)
Melatonin	Salinity					
0	0	15.33 ± 0.47 c	8.5 ± 0.35 c	23.83 ± 0.42 c	17.16 ± 0.84 e	79 ± 0.81 c
	150 mM	10.86 ± 0.59 ef	5.85 ± 0.35 e	16.71 ± 0.81 g	12.66 ± 0.23 g	59.66 ± 1.24 e
	300 mM	6.1 ± 0.04 g	3.75 ± 0.25 f	9.85 ± 0.3 h	7.83 ± 0.23 i	37.66 ± 1.69 h
30 ppm	0	18.13 ± 0.42 b	10.75 ± 0.35 b	28.88 ± 0.81 b	21.2 ± 0.57 c	81.66 ± 0.47 b
	150 mM	15.66 ± 0.47 c	9.12 ± 0.35 c	24.79 ± 0.29 c	18.66 ± 0.47 d	70.66 ± 0.94 d
	300 mM	11.66 ± 0.47 e	7.25 ± 0.35 d	18.91 ± 0.82 f	14.66 ± 0.47 f	53.66 ± 1.24 f
60 ppm	0	20.95 ± 0.04 a	12.03 ± 0.25 a	32.98 ± 0.21 a	25.16 ± 0.62 a	86.33 ± 1.24 a
	150 mM	18.33 ± 0.44 b	10.75 ± 0.27 b	29.08 ± 0.82 b	23.83 ± 0.47 b	77 ± 0.81 c
	300 mM	14.16 ± 0.23 d	8.5 ± 0.35 c	22.66 ± 0.31 d	17.33 ± 0.47 e	58.66 ± 0.94 e
90 ppm	0	16 ± 0.42 c	8.75 ± 0.17 c	24.75 ± 0.39 c	17.86 ± 0.47 de	79 ± 0.94 c
	150 mM	13.66 ± 0.44 d	7.52 ± 0.37 d	21.15 ± 0.3 e	15.33 ± 0.23 f	70.33 ± 1.24 d
	300 mM	10.66 ± 0.47 f	6.5 ± 0.35 e	17.16 ± 0.42 g	10.66 ± 0.47 h	44.66 ± 1.24 g

The highest chlorophyll a, chlorophyll b, total chlorophyll, carotenoid, and relative water contents were 20.94, 12.03, 32.97, 25.16 mg g^−1^ FW, and 86.33%, respectively under 60 ppm of melatonin without salinity. Besides, the application of different levels of melatonin along with different concentrations of salinity not only significantly prevented (*p* < 0.01) the reduction in the contents of these traits, but also enhanced their contents in relation to salinity treatment. The results showed that applying 60 ppm of melatonin to salinity-treated fenugreek plants (300 mM) effectively enhanced (*p* < 0.01) chlorophyll a (132%), chlorophyll b (126%), total chlorophyll (130%), carotenoid (121%), and relative water (56%) contents compared with salinity treatment without melatonin application (Table 1).

### The effects of melatonin on antioxidant system under salt stress

Salinity stress, especially at the level of 300 mM, significantly raised (*p* < 0.01) the soluble protein content compared to the control. Application of 60 ppm melatonin in the absence of salinity substantially enhanced the soluble protein content by 40% compared to the control. Besides, the combination of varied salinity levels and melatonin led to a considerable increase (*p* < 0.01) in the amount of soluble protein compared to the salinity stress treatment. The greatest soluble protein content reached 518.33 mg ml^−1^ protein FW when 300 mM of salinity was paired with 60 ppm of melatonin, indicating a 298% increase in soluble protein content compared to the control (Table 2).

**Table 2:** The mean comparison effects of melatonin concentrations and different levels of NaCl on soluble protein content and antioxidant enzyme activities.

Treatment		Soluble protein (mg ml^−1^ protein)	Catalase (nmol of H_2_O_2_ decomposed min^−1^ mg^−1^ protein)	Polyphenol oxidase (nmol/min^−1^ mg^−1^ protein)	Guaiacol peroxidase (nmol guaiacol min^−1^ mg^−1^ protein)	Ascorbate peroxidase (nmol oxidized ascorbate min^−1^ mg^−1^ protein)	Superoxide dismutase (nmol/min^−1^ mg^−1^ protein)
Melatonin	Salinity						
0	0	130 ± 4.08 g	0.02 ± 0.002 f	0.03 ± 0.002 fg	0.04 ± 0.002 f	0.04 ± 0.003 k	0.03 ± 0.002 e
	150 mM	265.33 ± 13.42 e	0.03 ± 0.003 de	0.05 ± 0.0009 de	0.08 ± 0.001 e	0.07 ± 0.001 h	0.05 ± 0.002 d
	300 mM	343.33 ± 9.42 d	0.04 ± 0.002 cd	0.06 ± 0.001 c	0.16 ± 0.001 c	0.1 ± 0.004 g	0.07 ± 0.003 b
30 ppm	0	154.33 ± 3.29 fg	0.025 ± 0.0009 ef	0.033 ± 0.0004 fg	0.042 ± 0.0004 f	0.05 ± 0.002 j	0.043 ± 0.001 e
	150 mM	389.33 ± 6.54 c	0.044 ± 0.003 c	0.06 ± 0.001 c	0.15 ± 0.008 c	0.16 ± 0.001 d	0.06 ± 0.004 c
	300 mM	440 ± 32.65 b	0.06 ± 0.002 b	0.07 ± 0.002 b	0.21 ± 0.005 b	0.2 ± 0.003 c	0.08 ± 0.0005 b
60 ppm	0	180 ± 8.16 f	0.035 ± 0.0004 d	0.04 ± 0.0008 f	0.05 ± 0.0009 f	0.06 ± 0.001 i	0.05 ± 0.002 d
	150 mM	456.66 ± 16.99 b	0.06 ± 0.005 b	0.07 ± 0.001 b	0.21 ± 0.008 b	0.21 ± 0.001 b	0.07 ± 0.004 b
	300 mM	518.33 ± 14.33 a	0.085 ± 0.006 a	0.08 ± 0.001 a	0.28 ± 0.001 a	0.24 ± 0.002 a	0.08 ± 0.001 a
90 ppm	0	140 ± 10.03 g	0.024 ± 0.001 ef	0.03 ± 0.0004 fg	0.048 ± 0.0008 f	0.04 ± 0.001 k	0.03 ± 0.001 ef
	150 mM	320 ± 8.16 d	0.04 ± 0.001 cd	0.04 ± 0.0009 de	0.13 ± 0.008 d	0.14 ± 0.003 f	0.05 ± 0.0003 d
	300 mM	406.66 ± 9.42 c	0.06 ± 0.008 b	0.05 ± 0.001 d	0.2 ± 0.004 b	0.15 ± 0.005 e	0.06 ± 0.0009 c

Compared to the control, catalase activity rose significantly (*p* < 0.01) as a function of the applied salinity level; whereas the application of different levels of melatonin had no significant effect on this parameter. Using different levels of melatonin along with different levels of salinity caused a very sharp rise in the activity of this enzyme. The highest activity of catalase reached 0.085 nmol of H_2_O_2_ decomposed min^−1^ mg^−1^protein by applying 60 ppm of melatonin and the salinity stress of 300 mM, which indicated a 325% increase in the activity of this enzyme compared to the control (Table 2).

The polyphenol oxidase activity under the salinity stress of 150 and 300 mM was enhanced (*p* < 0.01) by 66 and 100%, respectively, compared to the control; whereas the application of melatonin in the absence of salinity did not influence the activity of this enzyme. The highest polyphenol oxidase activity (0.08 nmol min^−1^ mg^−1^ protein); however, was observed in the treatment of 60 ppm melatonin and salinity of 300 mM (Table 1). Thus, using 30 and 60 ppm of melatonin with different salinity levels led to a substantial rise in polyphenol oxidase activity compared to the salinity treatment alone (Table 2).

Exposing the plants to salinity stress resulted in increased guaiacol peroxidase activity (*p* < 0.01). The application of different levels of melatonin; on the other hand, did not change the activity of this enzyme compared to the control. As far as, the addition of melatonin to the treatments came along with different salinity levels, a sharp rise in the activity of this enzyme was observed. The highest activity of guaiacol peroxidase (0.28 nmol guaiacol min^−1^ mg^−1^ protein) was recorded by applying 60 ppm of melatonin and the salinity stress of 300 mM (Table 2). The activity of this enzyme in the mentioned treatment increased by 600% compared to the control.

As a consequence of administering both salt stress and melatonin, ascorbate peroxidase activity significantly increased (*p* < 0.01) compared to the control. Additionally, simultaneous application of melatonin and salinity stress greatly increased the activity of this enzyme in comparison to salinity stress alone (without the use of melatonin). The highest activity of ascorbate peroxidase was 0.24 nmol oxidized ascorbate min^−1^ mg^−1^protein at salinity stress of 300 mM accompanied with 60 ppm of melatonin, which indicated a 500% increase in the activity of this enzyme compared to the control (Table 2).

Applying salinity stress notably raised (*p* < 0.01) the activity of the superoxide dismutase enzyme compared to the control. The activity of this enzyme under the salinity stress of 150 and 300 mM rose by 66 and 133%, respectively. Besides, a higher activity of superoxide dismutase (0.098 nmol min^−1^ mg^−1^ protein) was observed in the pots treated with 60 ppm of melatonin along with 300 mM of NaCl. Simultaneous application of 150 mM of NaCl and 30 and 60 ppm of melatonin as well as 300 mM of NaCl and 60 ppm of melatonin significantly increased the superoxide dismutase activity compared to the salinity stress alone (without the use of melatonin; Table 2).

### The effects of melatonin on oxidative stress under salt stress

The electrolyte leakage index and malondialdehyde content after using 300 mM of salinity were enhanced by 77 and 160%, respectively. The highest and lowest amount of these two indicators were observed in salinity stress of 300 mM without the use of melatonin and in the treatment of 60 ppm of melatonin (without salinity stress), respectively (Table 3). Treating the plants grown under salinity stress (300 mM) with different levels of melatonin (especially 60 ppm) significantly reduced electrolyte leakage index and malondialdehyde content by 40 and 55% compared to the salinity stress condition (without the use of melatonin).

**Table 3:** The mean comparison effects of melatonin concentrations and different levels of NaCl on malondialdehyde content, electrolyte leakage index, H_2_O_2_, and nitric oxide content.

Treatment		Electrolyte leakage (%)	Malondialdehyde (μ mol g^−1^ FW)	H_2_O_2_ content (μmol g^−1^ FW)	Nitric oxide content (nmol g^−1^ FW)
Melatonin	Salinity				
0	0	32.71 ± 0.7 hi	6.83 ± 0.23 e	13.5 ± 0.4 f	11.61 ± 0.35 f
	150 mM	46.12 ± 0.64 b	10 ± 0.81 b	20 ± 0.81 b	7.85 ± 0.16 h
	300 mM	58.05 ± 0.81 a	18.13 ± 0.65 a	27.66 ± 1.24 a	13.61 ± 0.2 de
30 ppm	0	33.30 ± 0.33 gh	6.720 ± 0.36 e	10.33 ± 0.47 g	17.2 ± 0.7 b
	150 mM	35.84 ± 09 ef	7.9 ± 0.29 d	14.96 ± 0.28 e	11.45 ± 0.38 f
	300 mM	39.21 ± 0.8 d	9.6 ± 0.32 c	17.26 ± 0.37 c	14.84 ± 0.32 c
60 ppm	0	31.64 ± 0.49 i	6.03 ± 0.04 e	9.13 ± 0.18 h	23.79 ± 1.07 a
	150 mM	33.47 ± 0.86 gh	6.33 ± 0.23 e	12.66 ± 0.47 f	12.87 ± 0.24 e
	300 mM	35.12 ± 1.45 fg	8.46 ± 0.36 d	16.33 ± 0.47 cd	14.04 ± 0.4 cd
90 ppm	0	34.45 ± 0.29 fgh	8 ± 0.23 d	13 ± 0.01 f	8.88 ± 0.4 g
	150 mM	37.29 ± 0.82 e	8.18 ± 0.22 d	15.83 ± 0.23 de	10.89 ± 0.4 f
	300 mM	41.91 ± 0.63 c	9.78 ± 0.31 c	19.2 ± 0.28 b	16.51 ± 0.24 b

This study’s results showed a remarkable rise (17%; *p* < 0.01) in nitric oxide content under the influence of salinity stress (300 mM) compared to the control. Applying 30 and 60 ppm of melatonin led to 43 and 91% increases in the content of this trait compared to the control. The highest nitric oxide content (24 nmol g^−1^ FW) was obtained when applying 60 ppm of melatonin (Table 3). The combined use of different levels of melatonin and salinity stress (150 mM) considerably raised the nitric oxide content compared to the salinity treatment alone (150 mM without the use of melatonin).

The content of H_2_O_2_ rose (*p* < 0.01) remarkably following the application of different levels of salinity stress compared to the control. H_2_O_2_ content rose by 66 and 125% compared to the control after applying 150 and 300 mM of salinity, respectively. The use of different levels of melatonin significantly reduced the hydrogen peroxide content compared to the control. Moreover, using melatonin in plants treated with salinity significantly increased the content of this trait compared to plants treated with salinity alone. The highest amount of hydrogen peroxide was (28 μmol g^−1^ FW) recorded in 300 mM salinity treatment without the use of melatonin (Table 3).

### The effects of melatonin on non-enzymatic antioxidant contents and sugar accumulation under salt stress

**Figure 2:**
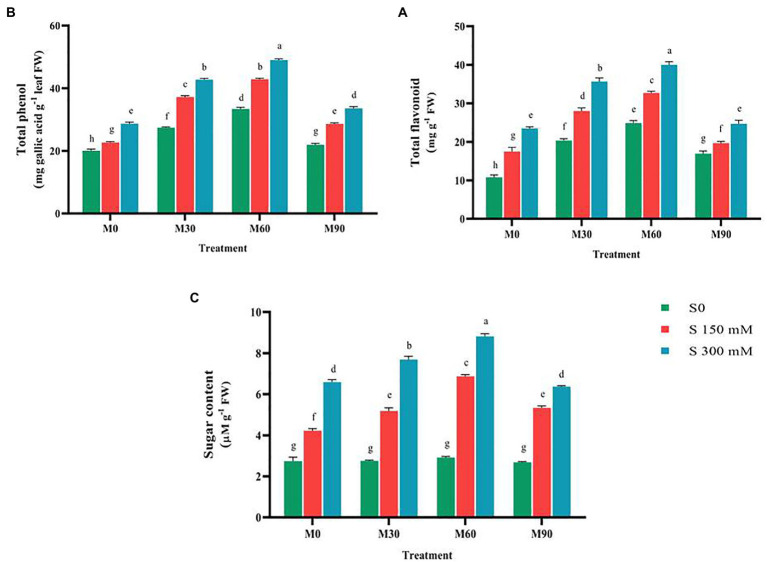
The mean comparison effects of melatonin concentrations and different levels of NaCl on **(A)** Total flavonoid, **(B)** Total phenol, **(C)** Sugar content. Duncan method at 1% probability level was applied to compare mean values. The columns having similar letters had no significant difference.

The flavonoid and total phenol content increased notably (*p* < 0.01) compared to the control as a function of the applied salinity levels. Moreover, applying different levels of melatonin without salinity enhanced these two traits compared to the control (Figure 2). Using 60 ppm of melatonin, the flavonoid and total phenol contents were enhanced by 133 and 75%, respectively. Different concentrations of melatonin applied to salt-stressed plants led to an increase in flavonoid and total phenol content compared to salinity stress conditions. The maximum levels of flavonoid and total phenol (40 and 46 mg g^−1^ FW) were seen when a salinity stress of 300 mM and 60 ppm of melatonin were simultaneously applied, however the levels of these compounds decreased dramatically when the melatonin dosage was increased to 90 ppm (Figure 2).

The salinity stress in a concentration-dependent manner caused a large increase (*p* < 0.01) in total sugar content compared to the control. This trait content, after using 150 and 300 mM of salinity, rose by 48 and 160%, respectively. Treating the plants with different levels of melatonin brought no significant change in total sugar content. Furthermore, the combined use of different levels of salinity and melatonin raised the total sugar content compared to the salinity treatments (without melatonin). The highest value (8.5 μM g^−1^ FW) of this trait was recorded under salinity treatment of 300 mM and melatonin level of 60 ppm, which indicated a 290% increase in the total sugar content compared to the control (Figure 2).

### The effects of melatonin on hormonal profile contents under salt stress

**Figure 3:**
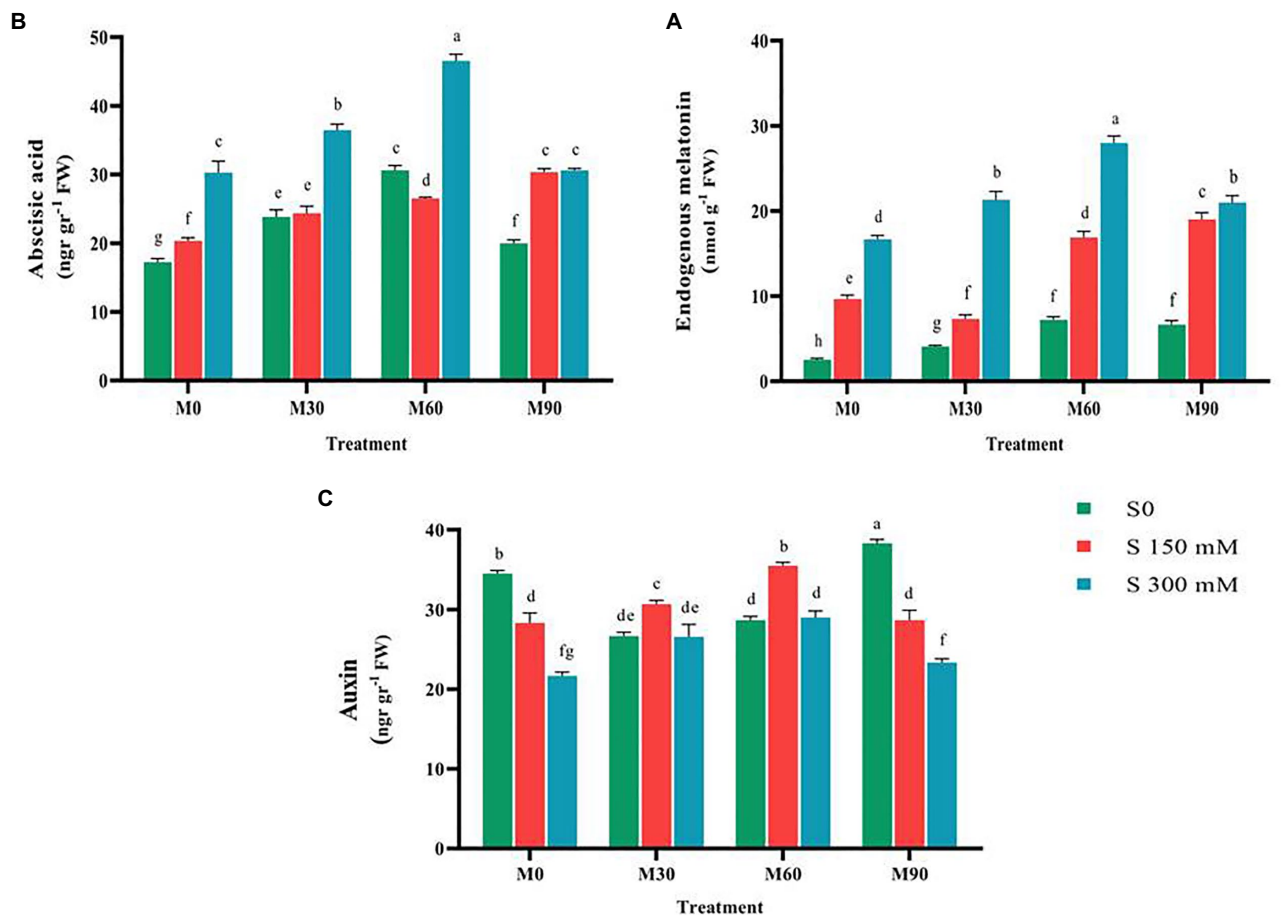
The mean comparison effects of melatonin concentrations and different levels of NaCl on **(A)** Endogenous melatonin content, **(B)** Abscisic acid content, **(C)** Auxin content. Duncan method at 1% probability level was applied to compare mean values. The columns having similar letters had no significant difference.

Compared to the control, endogenous melatonin content rose (*p* < 0.01) significantly under the effect of various degrees of salinity stress, with the changes being regulated by the administered salinity levels. Endogenous melatonin concentration was increased by 250 and 500% at salinity levels of 150 and 300 mM, respectively. Treating the plants with melatonin enhanced the endogenous melatonin content in plants compared to the control. The endogenous melatonin content after the use of 30, 60 and 90 ppm of melatonin reached to 4, 7 and 6.5 nmol g^−1^ FW. Salinity stress accompanied with different levels of melatonin raised the content of endogenous melatonin compared to the salinity stress alone. The highest content of endogenous melatonin (28 nmol g^−1^ FW) was observed in salinity stress at 300 mM and melatonin at 60 ppm (Figure 3), which indicated a 760% increase in the total sugar content compared to the control.

A review of the relevant researches showed that the effects of exogenous melatonin on the content of abscisic acid and auxin are quite different, as contradictory results have been reported (Arnao and Hernández-Ruiz, 2018, 2020; Zhang et al., 2019). Therefore, in this experiment, the relationship between melatonin and these two hormones under the control condition and salinity stress was investigated. The results of this study showed that salinity stress in a concentration-dependent manner has led to an increase (*p* < 0.01) in the content of abscisic acid (compared to the control). Compared to the control, the content of abscisic acid increased by 17 and 53% with the application of 150 and 300 mM of salinity, respectively. Thus, applying melatonin without salinity stress raised the content of this trait compared to the control. Simultaneous application of different levels of melatonin and salinity stress, especially at the salinity level of 300 mM and melatonin 60 ppm, significantly increased the content of abscisic acid (50%) in relation to salinity stress treatment (without the use of melatonin; Figure 3). In the mentioned treatment, the highest content of abscisic acid reached 45 ng g^−1^ FW.

When the average of various levels of melatonin and salinity stress were evaluated, the auxin content under the salinity stress was found to be significantly lower (*p* < 0.01) than in the control. When 150 and 300 mM of salt were applied, the auxin content fell by 25 and 66%, respectively, compared to the control. Indeed, the severity of applied salinity stress was a factor of influence in lowering the auxin content. The combined use of different levels of melatonin and salinity stress considerably raised the auxin content compared to the salinity treatment alone without the use of melatonin (Figure 3). Auxin content increased 30% by applying 60 ppm of melatonin and the salinity stress of 150 mM compared to the salinity treatment alone.

### The effects of melatonin on mineral element contents under salt stress

**Figure 4:**
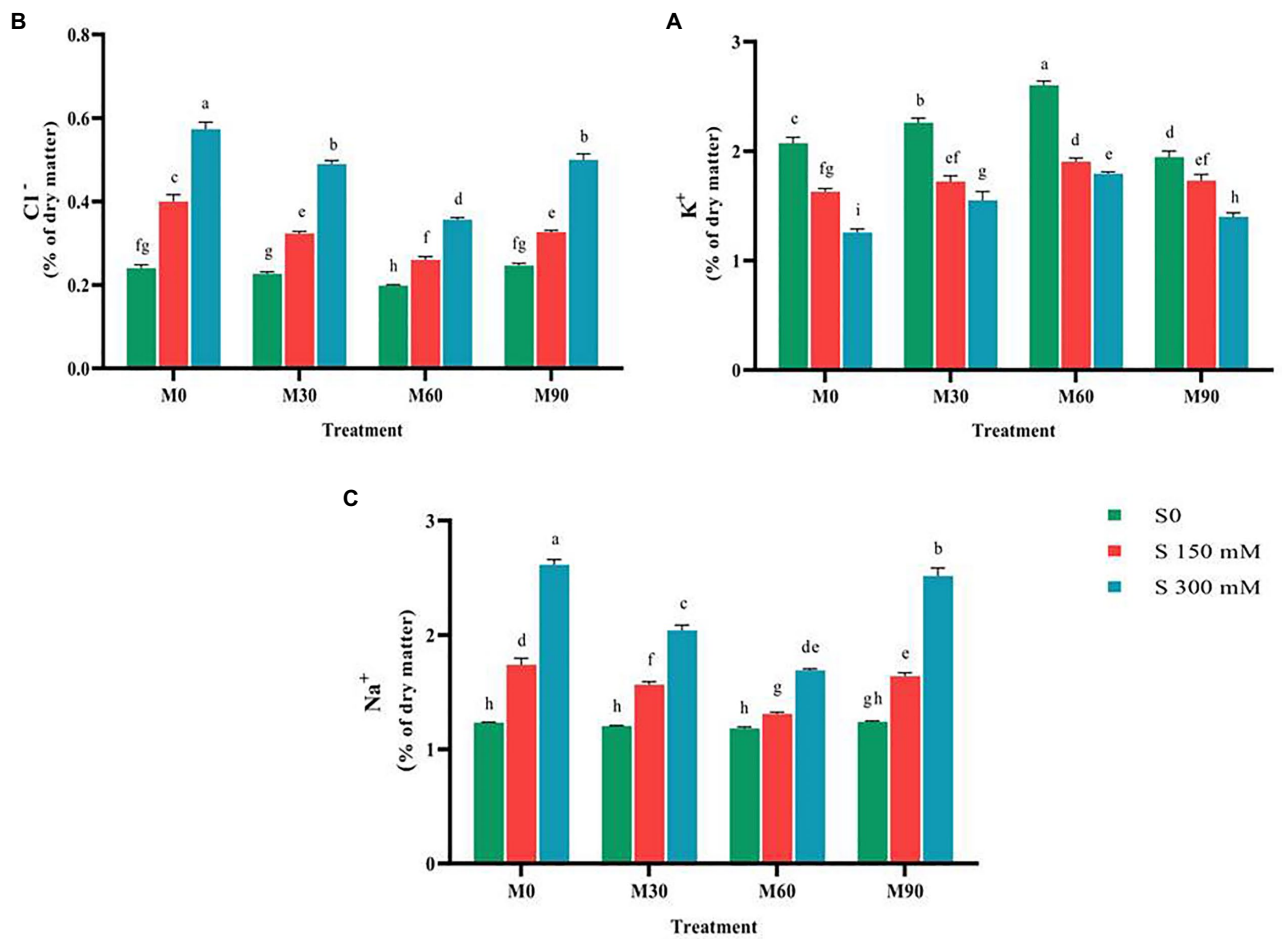
The mean comparison effects of melatonin concentrations and different levels of NaCl on **(A)** Potassium content, **(B)** Chlorine content, **(C)** Sodium content. Duncan method at 1% probability level was applied to compare mean values. The columns having similar letters had no significant difference.

The application of salinity stress significantly decreased (*p* < 0.01) the potassium content relative to the control. In contrast, exposing plants to varying concentrations of melatonin in the absence of salt stress led to a considerable increase in potassium content relative to the control. The potassium concentration increased by 20 and 50%, respectively, when treated with 30 and 60 ppm of melatonin. Simultaneous treatment of salinity stress and different levels of melatonin led to an increase in potassium content relative to salinity stress (no melatonin application). The highest potassium content (3% of dry matter) was achieved in the treatment of 60 ppm of melatonin and no salinity stress, and the lowest content (1.3% of dry matter) of this trait was recorded in the salinity stress of 300 mM (Figure 4).

After applying the salinity stress, performing a mean comparison of chlorine content revealed a rising trend (*p* < 0.01). Salinity stress of 150 and 300 mM resulted in 81 and 166% increase in chlorine content compared to the control. Application of 60 ppm melatonin without salinity stress remarkably reduced the chlorine content (15%) compared to the control; while other levels had no significant effect on this trait. Furthermore, simultaneous application of salinity stress at different levels of melatonin significantly lowered the chlorine (Cl^−^) content compared to the salinity stress treatment (without melatonin). It was observed that the application of 60 ppm of melatonin more effectively prevented the accumulation of chlorine in plant tissues under salinity stress (300 mM; Figure 4), so that by using this treatment, the chlorine content was reduced by 55% compared to the salinity treatment of 300 mM without the use of melatonin.

The salinity stress in a concentration-dependent manner led to a significant rise in intra-tissue sodium content (*p* < 0.01). Salinity stress of 150 and 300 mM resulted in a 50 and 100% increase in intra-tissue sodium content compared to the control. Treating the plants with different levels of melatonin (in absence of salinity stress) had negligible effects on sodium content (Na^+^) compared to the control. The combined use of melatonin and salinity considerably reduced the sodium content in plants compared to the salinity stress treatment (no melatonin application; Figure 4). The sodium content after using 60 ppm melatonin along with 300 mM of salinity decreased by 60% compared to the salinity treatment alone (no melatonin application).

### The effects of melatonin on diosgenin biosynthesis pathway genes expression and diosgenin content

**Figure 5:**
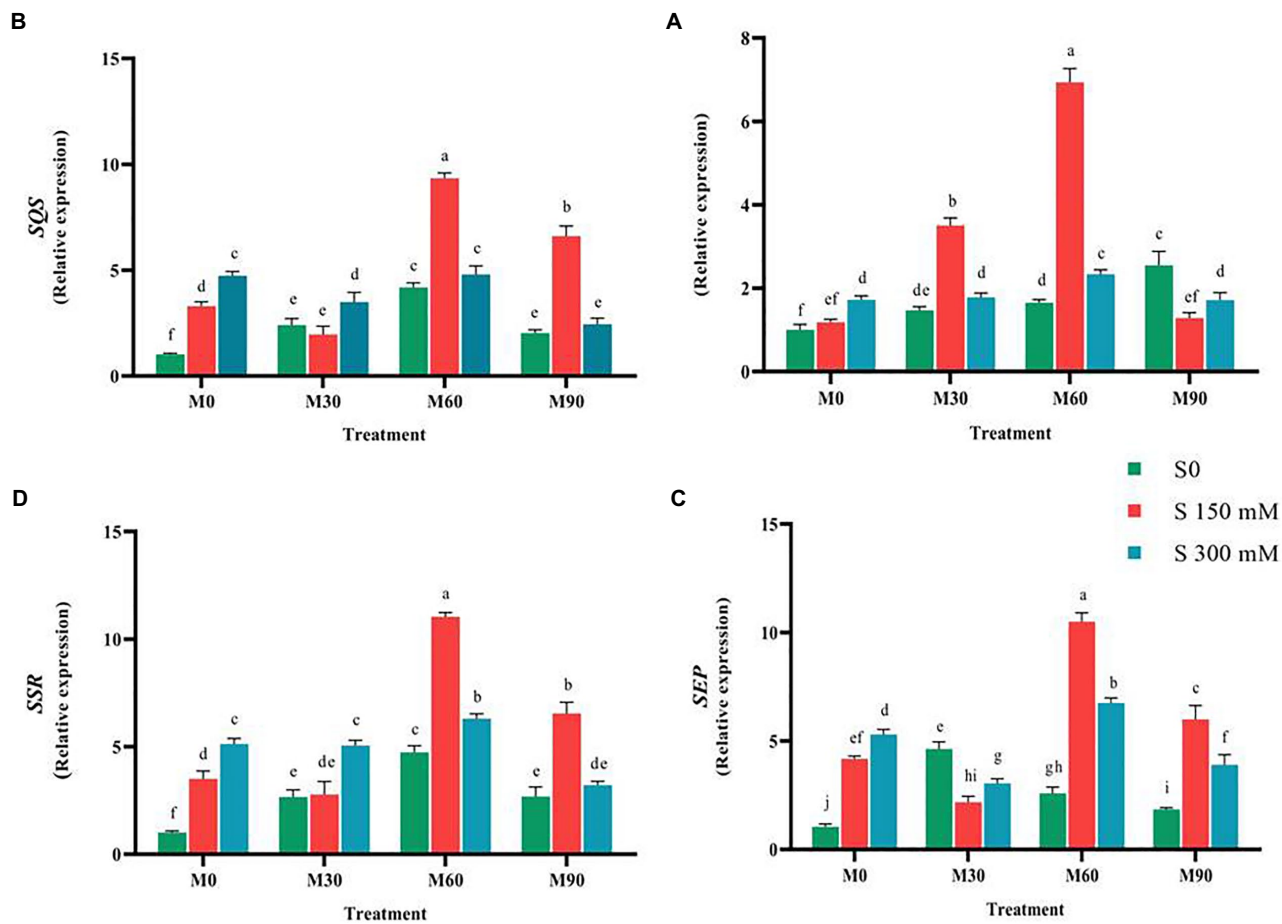
The mean comparison effects of melatonin concentrations and different levels of NaCl on the expression of **(A)** *CAS*, **(B)** *SQS*, **(C)** *SEP*, **(D)** *SSR*. Duncan method at 1% probability level was applied to compare mean values. The columns having similar letters had no significant difference.

The expression of *CAS* (Cycloartenol synthase) gene under the influence of 150 mM salinity level (without melatonin) was not significantly different from the control, while it considerably increased (two-fold) at the level of 300 mM (Figures 1, 5). The expression of this gene sharply increased (*p* < 0.01) under the influence of different levels of melatonin (without salinity) so that it increased three-fold in the plants treated with 90 ppm of melatonin. Under salinity stress of 150 mM along with 60 ppm of melatonin, the expression of this gene increased seven times compared to the control. *CAS* gene expression was notably reduced when the plants were exposed to 300 mM salinity and treated with different levels of melatonin compared to the 150 mM NaCl. Furthermore, the expression of this gene was higher in 150 mM NaCl along with 60 and 30 ppm melatonin levels than in other treatments (Figure 5).

Compared to the control, salt and melatonin treatment increased (*p* < 0.01) *SQS* gene (squalene synthase) expression. In compared to the salinity stress, simultaneous use of salinity (150 mM) and melatonin (60 and 90 ppm) greatly enhanced the expression of this gene (without melatonin), but simultaneous use of salinity (300 mM) and melatonin (60 ppm) had no significant influence on the expression of this gene. In the presence of 150 mM salinity and 60 ppm melatonin, the greatest expression of *SQS* (nine-fold) was observed (Figure 5).

Applying salinity stress (without the use of melatonin) in a concentration-dependent manner substantially enhanced the expression of *SEP* gene compared to the control. In comparison with the control, the expression of *SEP* rose significantly (4 and 5.3-fold increase, respectively, *p* < 0.01). Treating the fenugreek plants with low melatonin concentration (30 ppm) did not change the expression of this gene compared to the salinity stress (without the use of melatonin), while the expression of this gene underwent an increase under higher melatonin concentrations (60 and 90 ppm). The combined use of salinity stress (150 mM) and melatonin (60 and 90 ppm) enhanced the expression of this gene relative to the salinity stress (without melatonin use), while simultaneous application of salinity stress (150 mM) and melatonin (30 ppm) resulted in a significant decrease in the expression of this gene relative to the salinity stress. The simultaneous application of salinity stress (300 mM) and melatonin (30 and 90 ppm) and salinity stress (300 mM) and melatonin (60 ppm) decreased and increased the expression of this gene compared to salinity stress (without melatonin), respectively (Figure 5).

**Figure 6:**
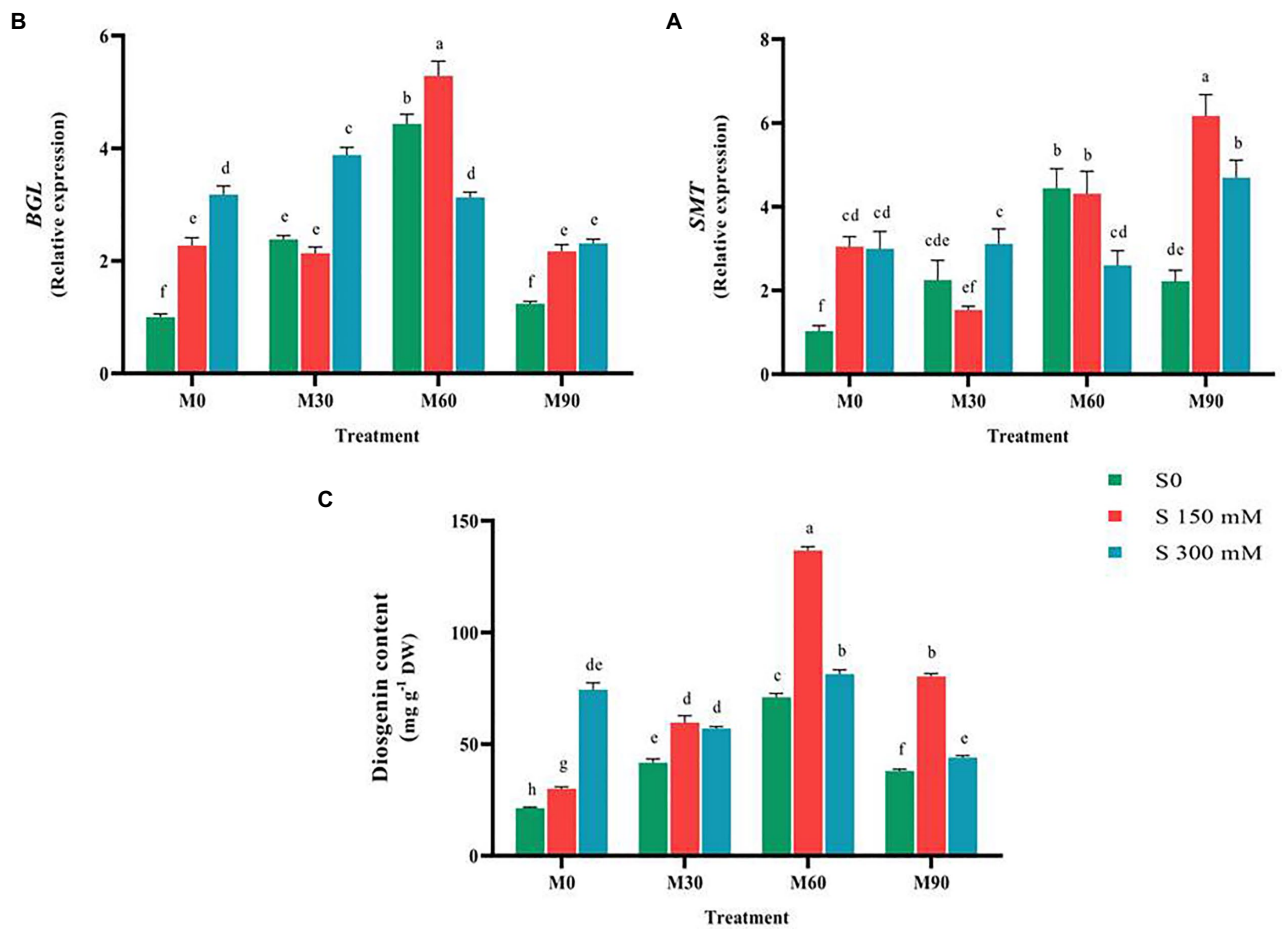
The mean comparison effects of melatonin concentrations and different levels of NaCl on the expression of **(A)** *SMT*, **(B)** *BGL*, and **(C)** Diosgenin content. Duncan method at 1% probability level was applied to compare mean values. The columns having similar letters had no significant difference.

Exposing the plants to melatonin and salinity resulted in a significant rise (*p* < 0.01) in the expression of *SSR* compared to the control. Applying 150 and 300 mM of salinity caused a significant increase (3.5 and 5.5-fold increase, respectively, *p* < 0.01) in the expression of *SSR* compared to the control. The highest expression of this gene (12-fold) was recorded when 60 ppm of melatonin was combined with salinity stress of 150 mM. When a salinity of 150 mM was combined with 30 ppm of melatonin, the expression of this gene was reduced (without the use of melatonin), whereas when the same salinity level was combined with 60 and 90 ppm of melatonin, the expression of this gene increased significantly 3.14 and 1.85-fold, respectively, compared to the salinity stress without melatonin (Figure 5). The application of salinity and melatonin notably raised (*p* < 0.01) the expression of *SMT* gene (sterol methyltransferase) compared to the control, although no significant difference was observed among different levels of salinity stress. The expression of *SMT* experienced 3.1 and 3-fold increases with the application of 150 and 300 mM of salinity, respectively, compared to the control. Furthermore, the expression of this gene under 30, 60 and 90 ppm of melatonin without salinity stress rose by 2.5, 4.5 and 2.2-fold, respectively. The highest expression of *SMT* (six-fold), however, was recorded in 150 mM of salinity treatment along with 90 ppm of melatonin (Figure 6).

*BGL* gene (26-O-Beta glucosidase) expression rose significantly (*p* < 0.01) under salinity stress without the use of melatonin compared to the control. Thus, the expression of this gene increased after using different levels of melatonin by 5 times (at the level of 60 ppm melatonin) compared to the control. The highest expression of this gene was recorded in the treatment of 150 mM NaCl combined with 60 ppm of melatonin by five-times compared to the control. Application of different levels of melatonin along with salinity stress of 300 mM significantly increased the expression of this gene compared to the control. The results showed that the interactions of salinity stress with different levels of melatonin raised the expression of this gene as a suitable stimulus. By increasing salinity stress from 150 to 300 mM (at the melatonin concentration of 60 ppm), the expression of this gene decreased (Figure 6).

Compared to the control, exposing the plants to salt and melatonin, particularly 60 ppm, dramatically increased (*p* < 0.01) the diosgenin level in fenugreek. When 150 and 300 mM of salt were applied, the diosgenin content increased significantly (50 and 275%, respectively) compared to the control. In addition, the diosgenin content rose by 100, 200 and 80% with the application of 30, 60 and 90 ppm of melatonin, respectively, compared to the control. The combined use of different levels of salinity (150 mM) and melatonin (all of the applied concentrations) substantially raised the diosgenin content compared to the 150 mM salinity treatment (without melatonin). Simultaneous application of different levels of salinity and melatonin (300 mM and 60 ppm) resulted in an increase in diosgenin content compared to the 300 mM salinity treatment (without melatonin). The highest content of diosgenin (131 mg g^−1^ DW) was recorded in the treatment of 150 mM salinity stress and 60 ppm of melatonin. By increasing salinity stress from 150 to 300 mM and melatonin concentration from 60 to 90 ppm, the diosgenin content considerably fell compared to NaCl of 150 mM (Figure 6).

## Discussion

### Exogenous melatonin alleviated NaCl-induced inhibition in biosynthesis of plant pigments and relative water content under salt stress

Given the fact that plant pigments such as chlorophylls and carotenoids, as the most important factors involved in photosynthesis, are considerably impaired by environmental stresses, monitoring these compounds may well highlight the impacts of any specific stresses on plants (Siddiqui et al., 2020a). The results of this study showed that applying the different levels of salinity stress led to a decrease in chlorophylls, carotenoids, and relative water contents in fenugreek. Our findings revealed that the application of melatonin (especially the level of 60 ppm) under the control (without salinity stress) and salinity condition enhanced (*p* < 0.01) the content of chlorophylls, carotenoids, and relative water (Table 1). Melatonin stimulates the synthesis of pigments by improving the genes involved in the biosynthesis pathway of chlorophylls and carotenoids (Sharif et al., 2018; Siddiqui et al., 2020a,b). The findings of this research back with earlier studies concerning melatonin’s beneficial effects on crops under the influence of abiotic stressors (Hossain et al., 2020; Ren et al., 2020). In fact, melatonin prevents the degradation of chlorophyll by enzymes such as chlorophyllase, phenophytinase, peroxidase, and regulates the enzyme pheophorbide *a* oxygenase (PaO; Sharif et al., 2018). The previous results suggest that the melatonin impact on photosynthetic pigments may be associated with increased capacity of the antioxidant system in plants (Li et al., 2017a; Xalxo and Keshavkant, 2019; Siddiqui et al., 2020a). In addition, the use of melatonin by increasing the concentration of magnesium ions, carotenoids, anthocyanins, and flavonoids, which are effective factors in photosynthesis, probably prevents the degradation of chlorophylls under stress (Li et al., 2017a; Bose and Howlader, 2020). In this study, melatonin increased the relative water content (RWC) of fenugreek plants subjected to salt stress to retain water (Table 1). It can be ascribed to the regulation of osmotic pressure by osmolytes or the improvement of water uptake by the roots (Keyvan, 2010). In fact, the exogenous application of melatonin enhances the photosynthetic activity and available plant water through osmotic regulation and reduction of H_2_O_2_ content in plants (Ren et al., 2020).

### Exogenous melatonin improved cellular redox homeostasis under salt stress

Under stress conditions, the content of reactive oxygen species and free radicals normally increases and the plant is exposed to oxidative damage. In this scenario, a variety of enzymatic and non-enzymatic antioxidant defense mechanisms might form in plants (Siddiqui et al., 2020b). The treatment of plants with different levels of melatonin (particularly 60 ppm) enhanced the enzymatic and non-enzymatic antioxidant defense mechanisms (compared to the control; Tables 2 and 3; Figure 2). Melatonin, by regulating and enhancing the expression of genes involved in physiological and biochemical processes, leads to increased plant tolerance to abiotic stresses (Mukherjee, 2019). Our finding proved that exogenous application of melatonin could improve fenugreek tolerance to salinity stress by increasing antioxidant protection by improving the biosynthesis of antioxidant enzymes. Various studies have confirmed the role of melatonin as an important antioxidant in enhancing the activity of antioxidant enzymes and the direct scavenging of H_2_O_2_ content under salinity (Zhang et al., 2014; Huang et al., 2017; Zeng et al., 2018), oxidative (Li et al., 2016), drought (Ye et al., 2016; Li et al., 2018), and cold (Yang et al., 2018b) stress conditions. In a study by Lee et al. (2017), exogenous application of melatonin reduced salinity-induced damage in rice by improving the activity of superoxide dismutase, catalase, and reducing the H_2_O_2_ content.

The results of transcriptome analysis confirmed that melatonin is a powerful antioxidant, which could enhance the biosynthesis of other antioxidants, and thus lead to the activation of defense mechanisms in plants (Weeda et al., 2014). In general, the results of this study implied that plant tolerance to salinity stress is largely improved by using the exogenous melatonin through stimulating the antioxidant defense system and modulating reactive oxygen species as well as accumulating soluble protein. It is well documented in previous studies that applying exogenous melatonin is distributed throughout the plasma membrane and it causes a considerable rise in endogenous melatonin content through messaging processes (Nawaz et al., 2016; Bose and Howlader, 2020). The formation of this process may result in decreased reactive oxygen species and H_2_O_2_ levels, and probably equilibrium in the cell by stimulating the genes closely related to the biosynthesis processes of enzymatic and non-enzymatic antioxidants (Arnao and Hernández-Ruiz, 2015; Sharif et al., 2018). The action of melatonin on enhancing the antioxidant enzyme activates and removing reactive oxygen species and free radicals is likely related to the decrease of electrolyte leakage index and malondialdehyde content following the administration of varied amounts of melatonin in this research (Zhang et al., 2020). The similar results about the effects of melatonin on the reduction of these two traits under abiotic stress have been reported in other plants (Li et al., 2017a; Zhang et al., 2020). It seems that melatonin by stimulating the expression of the genes closely associated with key metabolic pathways such as phenylpropanoid, chlorophylls, and carotenoids biosynthesis, carbon stabilization, and glucose metabolism will be able to effectively reduce the oxidative stress caused by salinity stress and improves fenugreek tolerance to this destructive stress (Arnao and Hernández-Ruiz, 2018; Bose and Howlader, 2020). Since phenol and flavonoid molecules have the ability to donate oxygen atoms to free radicals, they may scavenge free radicals (Arnao and Hernández-Ruiz, 2018; Sharif et al., 2018; Bose and Howlader, 2020).

Melatonin treatment (especially 60 ppm) considerably boosted the nitric oxide level of the plants in this research as compared to the control (Table 3). Various publications have reported on the beneficial benefits of salinity and melatonin on boosting nitric oxide concentration (Mukherjee, 2019). Increasing the concentration of endogenous melatonin after the application of exogenous melatonin and subsequent stimulation of nitric oxide biosynthesis is one of the first plant defense responses in signaling pathways, which ultimately leads to increased plant tolerance (Lee et al., 2017). The results of this study emphasize the role of nitric oxide as an important gaseous molecule with physiological functions including salinity stress tolerance. Even though the interactions of melatonin and nitric oxide under stress condition in plants are somewhat controversial, the current findings enhanced our insight into the relationship between melatonin and nitric oxide content under salinity stress in fenugreek (Lee et al., 2017). Interestingly, the neutralizing effects of antioxidants by melatonin are dependent on the production of nitric oxide under stress conditions. Therefore, the endogenous nitric oxide content improves after increasing stimulation by melatonin caused by salinity stress (Kaya et al., 2020).

The production of reactive oxygen species contributes in messaging activities on one hand, and on the other hand, if the reactive of oxygen concentration is higher than the desired level; it can lead to serious damage to the plant. The findings of this investigation revealed that the application of different levels of melatonin (30 and 60 ppm) under followed by a significant reduction in H_2_O_2_ content. Besides, treating the plants grown under salinity stress with melatonin (especially 60 ppm) successfully increased the H_2_O_2_ content compared to salinity treatment (no melatonin application; Table 3). The results of various studies show that melatonin effectively increases the expression of genes involved in antioxidant biosynthesis and thus considerably reduces H_2_O_2_ content (Lee et al., 2017). In general, the results of previous studies emphasize the role of melatonin in increasing salinity tolerance owing to its antioxidant properties and prevention of overproduction of reactive oxygen species. Melatonin induces salinity tolerance in plants by reducing the production of reactive oxygen species, lipid peroxidation, and inducing gene expression of various antioxidants (Kaya et al., 2020). Interestingly, in this study, the changes in fenugreek H_2_O_2_ and NO contents following the applied treatments were very similar, suggesting that these compounds play a prominent role in increasing plant tolerance under stress conditions. Damage to cell membranes due to cold stress may be assigned to the increase in malondialdehyde content and electrolyte leakage index, which in turn, may be arisen out of the elevated free radicals and H_2_O_2_ levels (Li et al., 2017b; Cao et al., 2018). Malondialdehyde and H_2_O_2_ contents considerably decreased following the use of melatonin, possibly due to the stimulation of biosynthesis of enzymatic and non-enzymatic antioxidants (Zhang et al., 2014; Huang et al., 2017; Zeng et al., 2018).

### Exogenous melatonin modulates endogenous hormone content

One of the valuable achievements of applying melatonin would be enhancement of the tolerance of plants to abiotic stresses *via* ABA-dependent pathway. It seems that abscisic acid is effective in increasing plant tolerance by regulating the content of reactive oxygen species (Arnao and Hernández-Ruiz, 2018, 2020; Sharif et al., 2018). The findings of this research showed that exogenous melatonin causes an increase in abscisic acid and endogenous melatonin content in In plants treated with salinity stress (Figure 3), which is likely regulated by melatonin’s direct and indirect effects on genes involved in intracellular chemical production (Arnao and Hernández-Ruiz, 2018, 2020; Sharif et al., 2018; Tiwari et al., 2020). Zhang et al. (2019) reported an increase in ABA biosynthesis and consequently a considerable improvement of plant tolerance to salinity stress. The results of various studies imply that the endogenous biosynthesis of melatonin increases under abiotic stress, which indicates the role of this hormone in plants responses to environmental stresses (Bose and Howlader, 2020). Accumulation of melatonin in plants is probably associated with enhanced expression of genes and activity of enzymes involved in melatonin biosynthesis. Exogenous melatonin infiltration enhances the plant’s endogenous melatonin content, which improves the plant’s resistance to salt stress (Bose and Howlader, 2020).

The role of auxin, as another regulator of plant growth, in abiotic stresses was proven (Arnao and Hernández-Ruiz, 2018; Sharif et al., 2018; Pérez-Llorca et al., 2019; Siddiqui et al., 2020a). The results of this study showed that the application of different levels of melatonin significantly enhanced the content of auxin, respectively (Figure 2). Changes in auxin content under salinity stress and melatonin treatment were reported in other studies (Pérez-Llorca et al., 2019). A positive and significant relationship between melatonin content and hormones, such as brassinosteroids (BRs), abscisic acid, and auxin was proven to regulate physiological processes in abiotic stress (Arnao and Hernández-Ruiz, 2018, 2020; Sharif et al., 2018; Pérez-Llorca et al., 2019; Siddiqui et al., 2020a). In this study, the increase of auxin content in plants treated with different levels of salinity and melatonin can be ascribed to increased expression of the main genes responsible for the biosynthesis of auxin and abscisic acid. Exogenous melatonin application possibly contributes in activating the H_2_O_2_, and NO-dependent pathway, enhancing the biosynthesis of abscisic acid and auxin, and regulating the signal transduction pathways, which raises fenugreek tolerance to abiotic stresses.

### Exogenous melatonin maintains ionic homeostasis

Our finding implied that the sodium (Na^+^) and chlorine (Cl^−^) content in fenugreek plants under salinity stress rose substantially in relation to the normal (Figure 4). The increased intra-tissue sodium is most likely to blame for the decrease in potassium absorption (Zahedi et al., 2020). Furthermore, owing to nutritional imbalance and the presence of high sodium and calcium in salinity, potassium absorption is hampered (Wang et al., 2013). The results of this study indicated that treating the fenugreek plant with different levels of melatonin, particularly 60 ppm, under salinity stress successfully raised the potassium content compared to salinity stress (without melatonin application; Figure 3). The application of melatonin could control the expression of ion channel genes and maintain ionic equilibrium in plant under salt stress *via* altering the activity of transcription factors involved in this process and as a result, ion homeostasis and plant tolerance to salinity stress generally rise (Zhao et al., 2018). Yu et al. (2018) reported that exogenous melatonin stimulated triacylglycerol breakdown, fatty acids β-oxidation, and energy turnover under salinity conditions, thereby contributing to the maintenance of plasma membrane H^+^–ATPase activity and K^+^/Na^+^ homeostasis in sweet potato. Reduced sodium and chlorine content due to different levels of melatonin may be associated with elevated Ca^2+^-ATPase activity in the plasma membrane, which provides the energy needed to remove large amounts of sodium (Liu et al., 2020).

### Exogenous melatonin effectively stimulates diosgenin biosynthesis pathway genes expression and diosgenin content

The effects of abiotic stresses on plants may result in negative changes in plant quality and quantity. Abiotic stresses effects in medicinal plants vary from those in other crops in that they may diminish biomass while increasing secondary metabolites. Therefore, the application of abiotic stresses in medicinal plants according to the purpose of their use (vegetative tissue or secondary metabolism) requires special expertise, particularly in identifying the intensity of this stress and the plant growth stage as well as using various stimulants (Parihar et al., 2015; Nxele et al., 2017). The concentration of stimulus and duration of exposure may highly contribute in increasing the performance of the desired stress and stimulus (Pavarini et al., 2012; Ebrahimibasabi et al., 2020). In this study, the combined effect of different levels of salinity stress and melatonin on the diosgenin content in fenugreek was evaluated to identify the best salinity and melatonin concentrations in order to achieve the highest diosgenin content.

The results of the present study indicated that salinity stress and melatonin application of 60 and 90 ppm have effectively enhanced the expression of the *SQS* gene, suggesting that this gene might be a pivotal point in the engineering of the diosgenin metabolite pathway (Figure 5). According to the results of the current study, similar to *SQS* gene salinity stress and the interaction of melatonin (60 ppm) and salinity stress resulted in the increased activity of *SEP,* and *CAS* genes compared to the control. Considering the favorable reaction of these two genes to melatonin, using of melatonin could be suggested as a suitable elicitor for the engineering of diosgenin. Since the activity of *CAS* gene is quite necessary in triggering the diosgenin biosynthetic pathway, its response to the interactions of salinity stress and melatonin promises identifying a suitable functional stimulus to increase the diosgenin content in fenugreek.

It is believed that there are some ambiguities in diosgenin biosynthetic pathway (Mohammadi et al., 2020). Moreover, exploring the complexities of this pathway and the functional elicitors with the aim of stimulating the increase of expression of these genes requires in-depth investigations. *SMT* and *SSR* genes expression increased under 150 mM and 300 mM of salinity compared to the control (Figures 5, 6). The two *SSR* and *SMT* genes compete for the biosynthesis pathway of diosgenin, with the *SSR* gene directing the pathway to diosgenin biosynthesis and the *SMT* gene directing the pathway to the biosynthesis of other plant sterols (Mohammadi et al., 2020; Zolfaghari et al., 2020). Therefore, this point is a very important point in the biosynthetic pathway of diosgenin.

The results of various studies show that stimulants such as salicylic acid, methyl jasmonates, brassinosteroids (BRs) alter and regulate the rate of biosynthesis, accumulation and exchange of substances stored in the vacuole, conversion or decomposition of plant metabolites, and eventually the amount of primary and secondary metabolites change (Odjakova and Hadjiivanova 2001). There are contradicting data about the application of various stimuli and the subsequent changes in the quantity of secondary metabolites, suggesting that different plants respond to stimuli in different ways. The length of stimulus exposure, development stage, and varying stimuli concentrations all influence plant responses (Angelova et al., 2006; Namdeo, 2007). Moreover, using melatonin in plants treated with salinity stress effectively enhanced the expression of the genes involved in the biosynthesis of diosgenin and ultimately the diosgenin content, while increasing the intensity of salinity stress (from 150 mM to 300 mM) along with melatonin (90 ppm) reduced the expression of all genes except for *SMT* gene. In fact, under conditions of severe (300 Mm of Nacl and 90 ppm of melatonin), increased expression of this gene leads the pathway of diosgenin biosynthesis to the production of other plant sterols, thereby increasing plant tolerance. Therefore, following the application of high melatonin concentration and severe salinity, which its destructive and toxic effects on plants were also proven in physiology section, the expression of *SMT* gene increased, which led to a decrease in the content of diosgenin. On the contrary, in lower doses of the applied treatments (150 Mm of Nacl and 30 and 60 ppm of melatonin), the expression of other genes involved in the pathway of diosgenin biosynthesis and finally the diosgenin content rose considerably compared to normal condition (Figure 6). Exogenous melatonin could effectively prevent the salt-induced accumulation of triacylglycerol in plant leaves (Yu et al., 2018). Furthermore, the genes responsible for fatty acid β-oxidation, triacylglycerol breakdown, and energy turnover are expressed more strongly following melatonin application which in turn leads to the accumulation of the main precursor of diosgenin biosynthesis, i.e., Acetyl-CoA. Accordingly, the higher diosgenin content in the plants treated by melatonin and encountered with salinity stress in the current study could be accounted for the same process. In general, the results of this study suggested that the combined use of salinity stress and melatonin effectively enhances the diosgenin content in fenugreek.

## Conclusion

This study suggested that exogenous application of melatonin, as an important functional stimulant, at the specific concentrations of 30 and 60 ppm, would trigger physiological and biochemical changes in salt-treated fenugreek in order to increase the plant tolerance to salinity stress. Melatonin, in fact, protects fenugreek from the negative effects of salt stress by increasing the manufacture of enzymatic and non-enzymatic antioxidants, chlorophylls, and diosgenin, as well as preserving ionic homeostasis. The primary results of this research may add to our understanding of the benefits of administering melatonin to plants that are stressed by the environment. In conclusion, the results of this study would provide a deep understanding of the melatonin signaling pathway in response to salinity-stressed fenugreek, which could be effective in breeding or biotechnology-based programs.

## Data availability statement

The raw data supporting the conclusions of this article will be made available by the authors, without undue reservation.

## Author contributions

AE: conceptualization, data curation, formal analysis, project administration, supervision, investigation and methodology, validation, visualization, writing—original draft, and writing—review and editing. MME: investigation and methodology. MRA and HA: validation, formal analysis, and review and editing. All authors contributed to the article and approved the submitted version.

## Conflict of interest

The authors declare that the research was conducted in the absence of any commercial or financial relationships that could be construed as a potential conflict of interest.

## Publisher’s note

All claims expressed in this article are solely those of the authors and do not necessarily represent those of their affiliated organizations, or those of the publisher, the editors and the reviewers. Any product that may be evaluated in this article, or claim that may be made by its manufacturer, is not guaranteed or endorsed by the publisher.

## Footnotes

^1^ http://eu.idtdna.com/calc/analyzer

^2^ https://www.ibm.com/products/spss-statistics
