# Supplementary material for: Melatonin confers fenugreek tolerance to salinity stress by stimulating the biosynthesis processes of enzymatic, non-enzymatic antioxidants, and diosgenin content
Source: Front Plant Sci. 2022 Aug 8;13:890613. doi: 10.3389/fpls.2022.890613 (PMC9394454; doi:10.3389/fpls.2022.890613)
Supplement: Supplementary file 1 [file Table_1.docx]

| **Annealing temperature** | **Length (bp)** | **Primer Sequence (′ 3 - ′5)** | | **Primer name** |
| --- | --- | --- | --- | --- |
|  |  | **Reverse** | **Forward** |  |
| 61 | 152 | CATTCTGTGTGTCTCCCTGCC | AGGTGGGAGATATGCTAGAATGGG | *SSR* |
| 62 | 140 | CACAGTAACTCATGACAACAGCAACCT | GAGAAGGCTGCAGAGGGTCTAG | *SMT* |
| 60 | 168 | CAGATGCACCAGAGAGTAATCG | CTATTCTCAGAAGGACCGATCTC | *SEP* |
| 60 | 17 | TACATGACGACGGTATTCTCCC | AAGAGAGATCCAACACCACTGC | *CAS* |
| 61 | 133 | TACCCACTGTTCCATTGCTATCC | GGCTCAACCATGATTCTCATACTG | *SQS* |
| 60 | 150 | CATCAGCCACACCTTGTCCTTC | CCTGGTCCTGAGAGCATAACAAATG | *BGL* |
| 61 | 230 | TATGTTTGTTGTTGGTGTCAACGAGCAAC | ATGTTAAATGATGCAGCCCTTCCACCT | *GAPDH* (Reference gene) |

**Table 1.** Primers used in this study and their characteristics
