# Supplementary material for: Melatonin confers fenugreek tolerance to salinity stress by stimulating the biosynthesis processes of enzymatic, non-enzymatic antioxidants, and diosgenin content
Source: Front Plant Sci. 2022 Aug 8;13:890613. doi: 10.3389/fpls.2022.890613 (PMC9394454; doi:10.3389/fpls.2022.890613)
Supplement: Supplementary file 2 [file Table_2.docx]

**Table S2.** Variance analysis of traits studied in this research (the experiment was performed as a factorial in a completely randomized design).

| Source of variance | df | Chlorophyll a | Chlorophyll b | Total chlorophyll | Carotenoids | Relative water content | Soluble protein | Catalase |
| --- | --- | --- | --- | --- | --- | --- | --- | --- |
| Salinity | 2 | 106.79** | 32.37** | 256.34** | 183.74** | 1413** | 121394** | 0.003** |
| Melatonin | 3 | 44.92** | 13.77** | 107.48** | 50.30** | 810.1** | 69250** | 0.001** |
| S * M | 6 | 32.26** | 11.61** | 82.16** | 56.41** | 453.4** | 26431** | 0.0004** |
| Error | 24 | 0.26 | 0.15 | 0.37 | 0.393 | 2 | 277.94 | 2.526e-05 |
| CV % |  | 3.58 | 4.73 | 2.71 | 3.72 | 2.12 | 5.34 | 10.94 |

| Source of variance | df | Polyphenol oxidase | Guaiacol peroxidase | Ascorbate peroxidase | Superoxide dismutase | Electrolyte leakage |
| --- | --- | --- | --- | --- | --- | --- |
| Salinity | 2 | 0.024** | 0.04** | 0.04** | 0.0012** | 228.02** |
| Melatonin | 3 | 0.0.23** | 0.02** | 0.13** | 0.0012** | 145.52** |
| S * M | 6 | 0.019** | 0.007** | 0.007** | 0.0005** | 161.3** |
| Error | 24 | 7.24e-05 | 3.701e-05 | 1.41 | 8.71e-06 | 0.92 |
| CV % |  | 10.32 | 4.41 | 3.007 | 4.76 | 2.51 |

| Source of variance | df | Malondialdehyde | Total flavonoid | Total Phenol | Sugar content | K^+^ | Na^+^ |
| --- | --- | --- | --- | --- | --- | --- | --- |
| Salinity | 2 | 49.62** | 439.8** | 589.8** | 31.82** | 0.54** | 1.26** |
| Melatonin | 3 | 36.31** | 227.7** | 228** | 15.77** | 0.49** | 0.54** |
| S * M | 6 | 24.48** | 129.5** | 153.5** | 6.45** | 0.32** | 0.66** |
| Error | 24 | 0.23 | 0.8 | 0.25 | 0.01 | 0.003 | 0.001 |
| CV % |  | 5.33 | 3.64 | 1.56 | 2.61 | 3.15 | 2.54 |

* and ** Significantly at the probability level of P <0.05 and P <0.01, respectively.

**Table S2** (continued)

| Source of variance | df | Cl^-^ | Na^+^/ K^+^ | Abscisic acid | Auxin | Nitric oxide content |
| --- | --- | --- | --- | --- | --- | --- |
| Salinity | 2 | 0.08** | 1.05** | 450.2** | 323.4** | 74.94** |
| Melatonin | 3 | 0.4** | 0.62** | 259.6** | 136.7** | 61.8** |
| S * M | 6 | 0.03** | 0.75** | 109.1** | 101.5** | 43.48** |
| Error | 24 | 0.0001 | 0.003 | 0.714 | 0.49 | 0.33 |
| CV % |  | 3.37 | 5.48 | 3.09 | 2.32 | 4.26 |

| Source of variance | df | Hydrogen peroxide | Melatonin content | Diosgenin | *SSR* | *SQS* | *SMT* | *SEP* | *CAS* | *BGL* |
| --- | --- | --- | --- | --- | --- | --- | --- | --- | --- | --- |
| Salinity | 2 | 102.84** | 829.1** | 3475** | 31.87** | 25.1** | 5.1** | 32.05** | 15.11** | 0.067** |
| Melatonin | 3 | 88.27** | 122.3** | 1024** | 10.48** | 6.7** | 10.14** | 10.69** | 1.96** | 0.489** |
| S * M | 6 | 58.83** | 26** | 3596** | 21.78** | 17.7** | 5.02** | 21.11** | 10.88** | 0201** |
| Error | 24 | 0.43 | 0.55 | 4.44e-05 | 0.17 | 0.14 | 0.21 | 0.15 | 0.19 | 0.006 |
| CV % |  | 4.18 | 5.57 | 3.44 | 9.24 | 9.75 | 14.48 | 9.1 | 12.7 | 12.89 |

* and ** Significantly at the probability level of P <0.05 and P <0.01, respectively.
